# A Flexible Calibration Method Using the Planar Target with a Square Pattern for Line Structured Light Vision System

**DOI:** 10.1371/journal.pone.0106911

**Published:** 2014-09-09

**Authors:** Qiucheng Sun, Yueqian Hou, Qingchang Tan, Guannan Li

**Affiliations:** 1 School of Mathematics, Changchun Normal University, Changchun, China; 2 College of Mechanical Science and Engineering, Changchun University, Changchun, China; 3 College of Mechanical Science and Engineering, Jilin University, Changchun, China; Xiamen University, China

## Abstract

A flexible calibration approach for line structured light vision system is proposed in this paper. Firstly a camera model is established by transforming the points from the 2D image plane to the world coordinate frame, and the intrinsic parameters of camera can be obtained accurately. Then a novel calibration method for structured light projector is presented by moving a planar target with a square pattern randomly, and the method mainly involves three steps: first, a simple linear model is proposed, by which the plane equation of the target at any orientations can be determined based on the square’s geometry information; second, the pixel coordinates of the light stripe center on the target images are extracted as the control points; finally, the points are projected into the camera coordinate frame with the help of the intrinsic parameters and the plane equations of the target, and the structured light plane can be determined by fitting these three-dimensional points. The experimental data show that the method has good repeatability and accuracy.

## Introduction

With the development of photo-electronics, image processing and electronics, computer vision technique is becoming increasingly relevant in industry for on-line inspection, component quality control, solid modeling and dimensional analysis [1]–[3]. Owing to the advantages of non-contact measurement, time efficiency, high flexibility and accuracy, line structured light techniques have found numerous applications [4]–[7]. A basic structured light vision system consists of a camera and a laser projector rigidly fixed with respect to the camera, and the working principle is laser triangulation. During the measurement, the projector projects light stripes on a measured object and the camera obtains images of the light stripes modulated by the depth of the object, then the 3D characteristic information of the measured surface can be acquired from the 2D deformed light stripe image.

The structured light vision system must firstly be fully calibrated before performing any 3D measurement. The goal of calibration is to establish the mapping relationship between the structured light plane and the computer image plane. The traditional approach for calibrating a structured light vision system incorporates two separate stages: camera calibration and projector calibration.

In the camera calibration stage, the intrinsic parameters and extrinsic parameters of camera model are usually calibrated by observing a calibration object whose geometry in 3D space is known with very good precision [8], [9]. Camera calibration can be done very efficiently by using Tsai’s method [10], in which an expensive calibration apparatus is usually required, e.g. a 3-D target. Zhang [11] proposed a flexible new technique for camera calibration by viewing a planar target from different unknown orientations. Accurate calibration points can be easily obtained using this method. Now, it is widely used in the camera calibration.

In the projector calibration stage, the coefficients of the structured light plane equation relative to the camera coordinate frame should be determined. Currently, different approaches for calibrating projector have been proposed in many literatures. In Robert Dewar’s method [12], several thin non-coplanar threads are strained in the space illuminated by a light stripe, and then several bright light dots are obtained as the control points whose 3D coordinates can be measured by means of a theodolite. However, this method requires complicated and expensive equipment, and its accuracy is affected by width restrictions of the light stripe. Huynh [13] has proposed a method, in which the world points on the light stripe plane are generated based on the invariance of the cross-ratio. In the method, a 3D calibration target usually consisting of two or three planes orthogonal to each other is needed for getting the control points, and it is difficult to be manufactured accurately. Recently, a planar target method has been proposed by Zhou [14], in which the intersection points of the light stripe and grids on the target are obtained as the control points. Since the quantity of the grids is limited, the number of control points is still not enough for calibrating projector accurately. Sometimes, to generate the intersection points of the grids and the light stripes, the orientation of the target is not flexible and random in the space. To make the system convenient to use on-site and under limited space, Wei [15] has proposed a novel 1D target-based calibration method for structured light vision sensors by randomly viewing a 1D target from different unknown orientations positioned within the field of view. Similar to Zhou’s method, the control points obtained are few in the method. Besides the above methods, there are other methods of calibrating a structured light vision system being presented in the literature [16]–[18]. But all these methods mainly have the following drawbacks: 1. it is difficult to generate large number of control points; 2. some methods require an elaborate and expensive calibration apparatus; 3. some methods are not suitable for field calibration.

In this paper, a novel approach for line structured light vision system calibration is proposed, in which a planar target with a square pattern is used. The proposed method only requires the camera to observe the planar target shown at a few (at least two) different orientations, and simultaneously the light stripe should be projected onto the target. Subpixel center localizations of the light stripe on the target are detected as the control points, which are sufficient to improve calibration accuracy. The paper is organized as follows. In the Method Section, a camera model is given first inspired by the work of Zhang, and then the detailed procedure of projector calibration is described, which includes working out the target plane, extracting center of the light stripe and fitting the structured light plane. Experimental studies are given in the next Section. The paper ends with conclusions.

## Methods

### 1. Calibration of camera model

The pinhole projective model was used to project 3D scenes onto the 2D camera image plane in Zhang’s method. The mapping between the world and image points proceeded through the four transformations, from world coordinates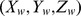, via camera coordinates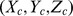, undistorted image coordinates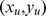, and distorted image coordinates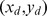, to pixel coordinates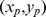, as shown in Table 1. Then, a nonlinear optimization function was established when the radial distortions were taken into consideration. The calibration was finished using the Levenberg–Marquardt (L–M) algorithm.

**Table 1:** Zhang’s calibration model.

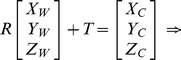 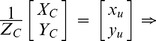 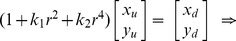 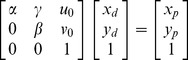
Optimization function: 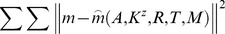
Where 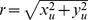 , 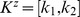

In the model, the optimization function aims at minimizing the distance error between the detected image point 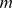 and the back-projected image point 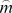 in 2D image pixels coordinate frame. However, the error criterion with respect to optimization and measurement is different while the measurement is implemented in 3D space. In other words, the equal pixel distance error in 2D image plane leads to diverse 3D metric distance error in different positions. The reasons mentioned above will cause accuracy decrease for 3D vision measurement [19]. Thus, the camera model is changed by minimizing the metric distance error between the projected points 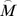 and the real points 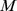 in 3D coordinate frame in this paper, and tangential distortion is also taken into consideration for improving accuracy, as shown in Table 2.

**Table 2:** The calibration model of the paper.

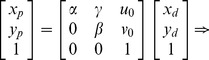 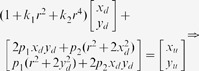 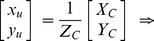 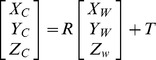
Optimization function: 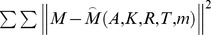
Where 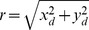

By means of the L-M algorithm, the model of this paper can be solved accurately. Thus, the intrinsic parameters of camera, the intrinsic matrix 
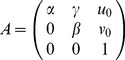
 and the distortion coefficient 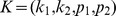, can be determined firstly.

### 2. Calibration of projector model

**Figure 1:**
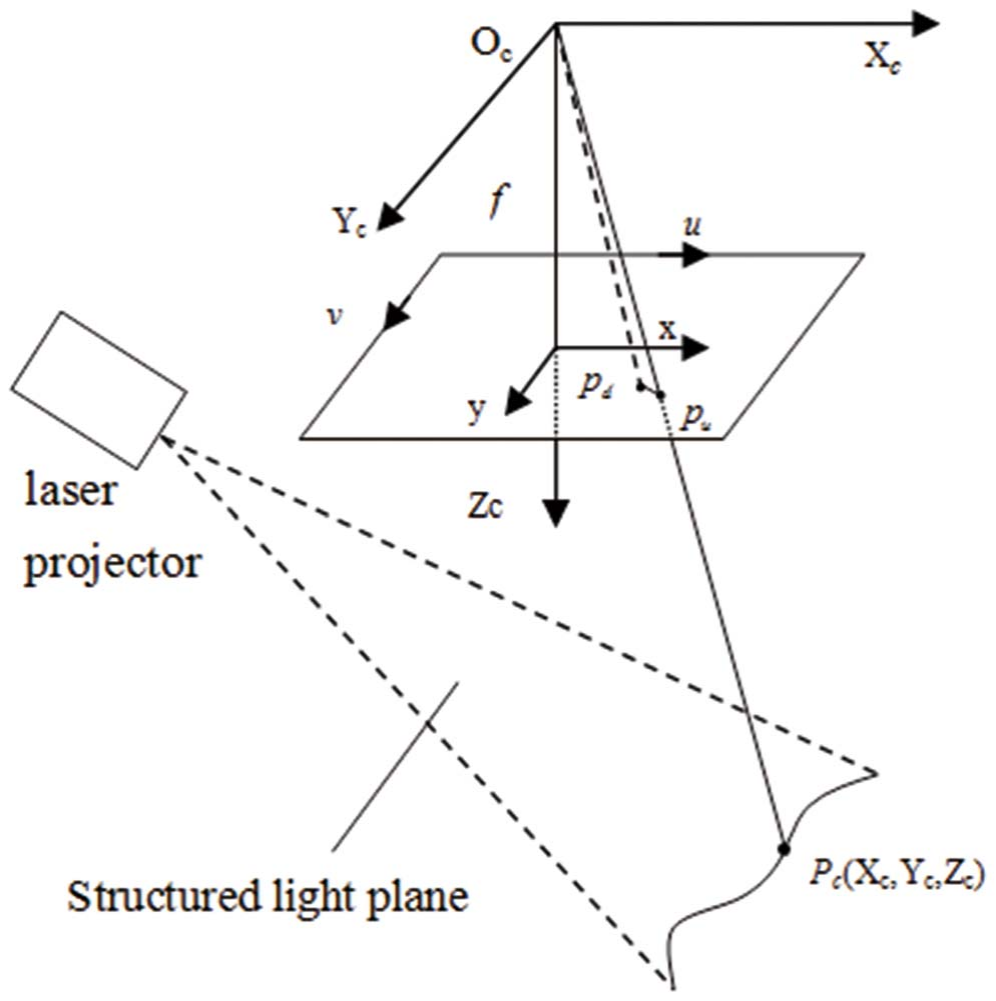
Overview of the structured light vision system.

It can be observed from Fig. 1 that the 3D point can’t be uniquely solved only by its known image point and the intrinsic parameters of camera model. Thus we need to determine the structured light plane, which is in the camera coordinate frame, to obtain the world coordinate from a known image point. It should be clarified that we assume that the camera coordinate frame is also the world coordinate frame. In this paper, a flexible method is given to determine the structured light plane, and the method mainly involves three steps: first, a linear algorithm is proposed to solve the plane equations of the target in the camera coordinate frame; second, the center points of the light stripe on the target are detected as control points, and these points are projected to the 3D space by means of the intrinsic parameters and the plane equations; finally, the structured light plane is determined by fitting the points in 3D.

#### 2.1 Determination of the target plane

**Figure 2:**
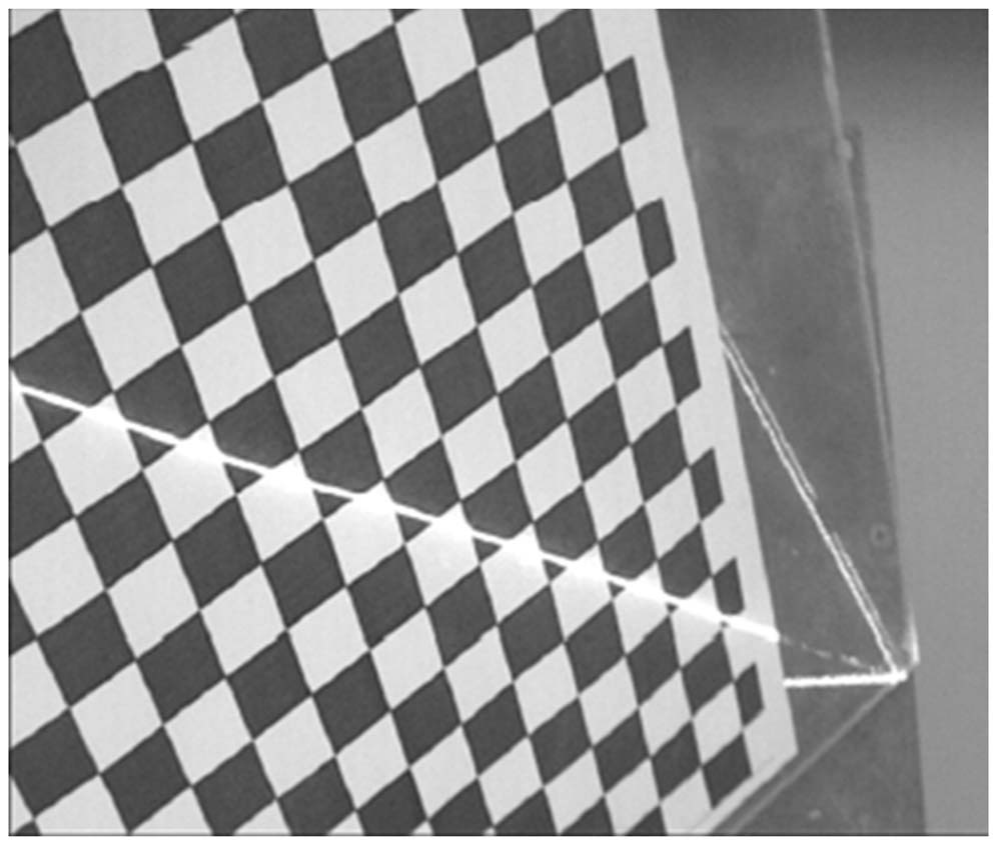
The imaging of light stripe on the checker board.

**Figure 3:**
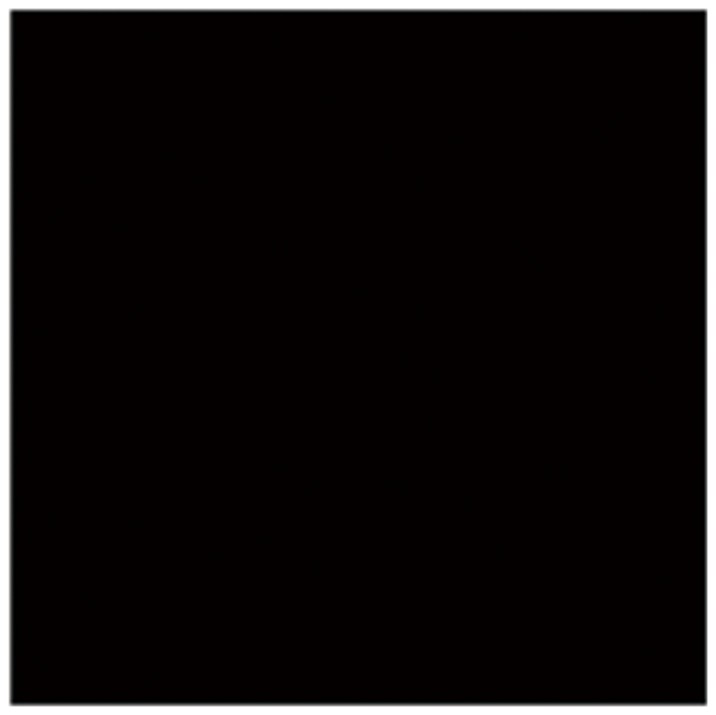
Target with a square pattern.

**Figure 4:**
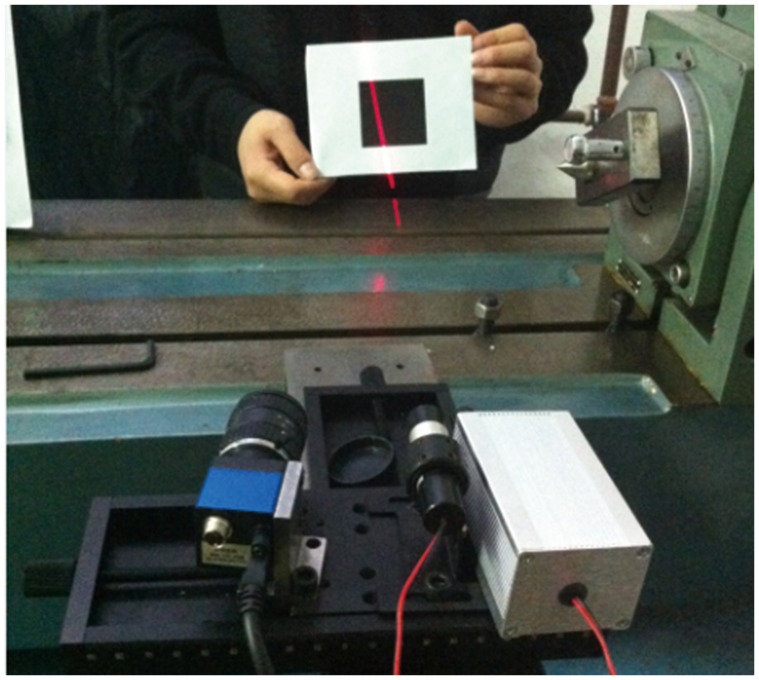
Calibration using the planar target.

Currently, the targets with checkerboard pattern are often used to calibrate the structured light plane. However, the imaging quality of the light stripe in the white areas of the targets is usually very poor, as shown in Fig. 2, which will lead to the loss of the detection precision of light strip center. Different from the previous targets, a planar target with a larger black square pattern, on which imaging quality of the light stripe can be guaranteed, is used in this paper, as shown in Fig. 3. When the camera and a light stripe projector are fixed together, some calibration patterns are acquired by means of the planar target shown at a few (at least two) different orientations, and the light stripe needs to be projected onto the target simultaneously, as shown in Fig. 4.

**Figure 5:**
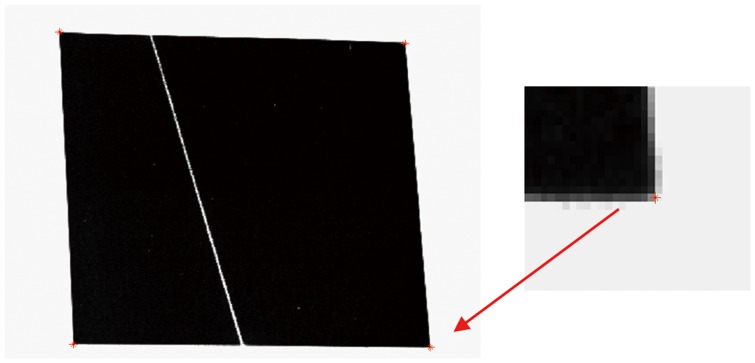
Corner points extraction.



To obtain the plane equation of the target, the image coordinates of four corner points in the pattern are firstly extracted using Bouguet’s method [20], as shown in Fig. 5. By using the image coordinates and the intrinsic parameters, the undistorted image coordinates 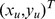 of the corner points can be solved, and the mapping from the undistorted image coordinates to the world coordinates 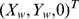 can be expressed as following:
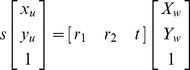
(1)where 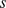 is an arbitrary scale factor, 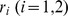 is the 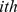 column vector of the rotation matrix, 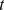 is the translation vector. Let 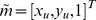, 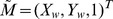, 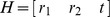, the equation (1) can be simplified as
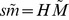
(2)dividing out *s* from Eq. (2),
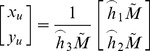
(3)where 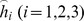 is the 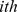 row of the matrix 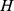.

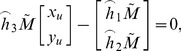
(4)

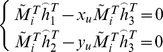
(5)


Then Eq. (5) can be rewritten as.
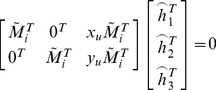
(6)


**Figure 6:**
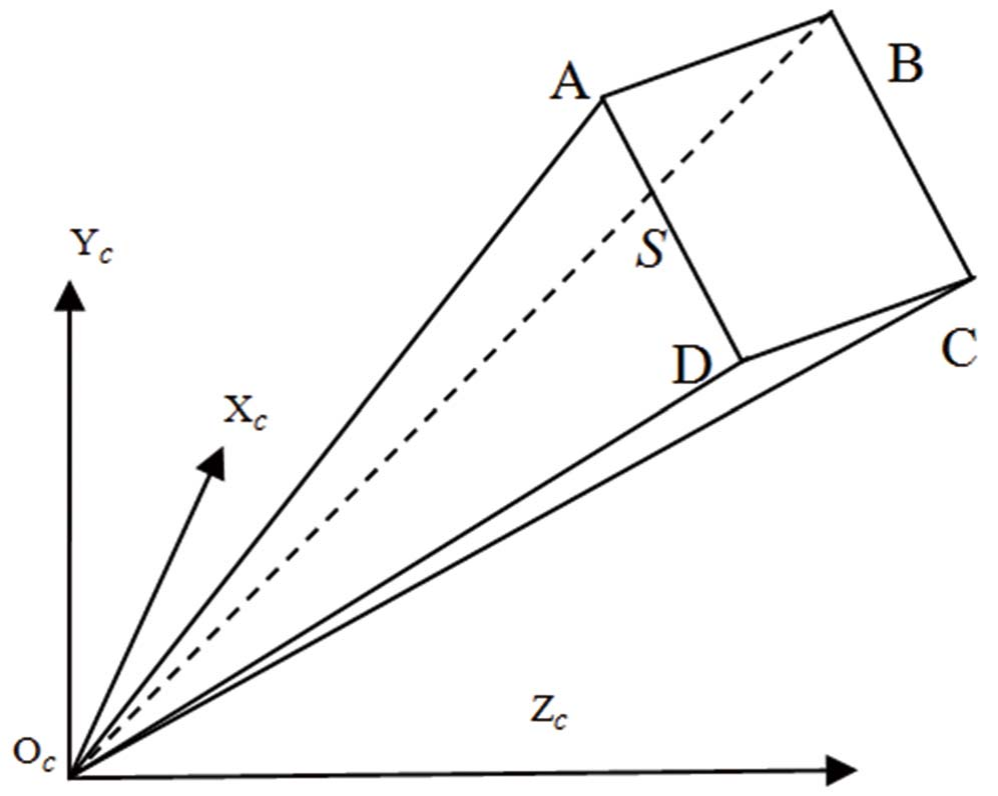
The target plane in camera coordinate frame.

Let 
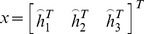
. When four points are given, we have four equations above, which can be written in matrix equation as 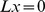, where *L* is a 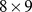 matrix. As *x* is defined up to a scale factor, the solution is well known to be the right singular vector of *L* associated with the smallest singular value. Using the rotation matrix and translation vector, the equation of the target plane can be solved easily in the camera coordinate system, as shown in Fig. 6.

#### 2.2 Subpixel center localization of the light stripe

Both in calibration and in measurement the center of light stripe is required. In previous works, there are many methods being proposed for extracting the light stripe center, and Steger’s method [21] is a representative one. The method extracts light stripe center based on the Hessian matrix of image intensity function at a pixel. It has high location precision, and has been widely used in vision measurement applications. However, due to multiple convolutions on the whole image, the method is slow and can not meet the requirements of real-time processing.

In this paper, we adopted a method presented in Reference [22] which combined Sobel and spatial-moment operator. Next we will show how this method works.

**Figure 7:**
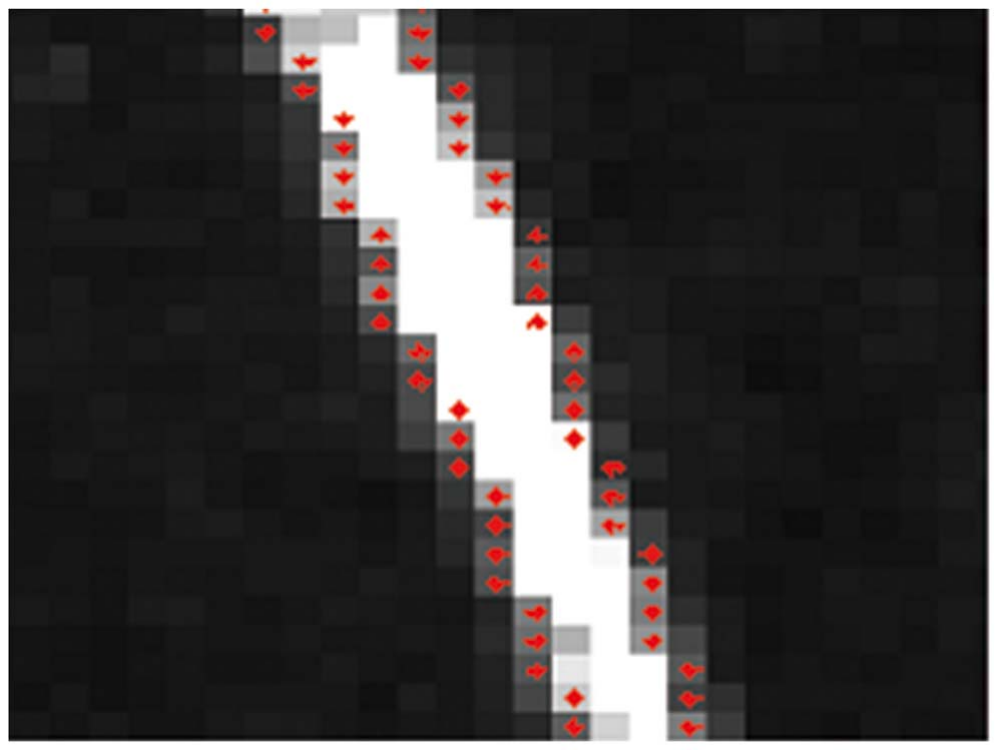
Edge points detection.

**Figure 8:**
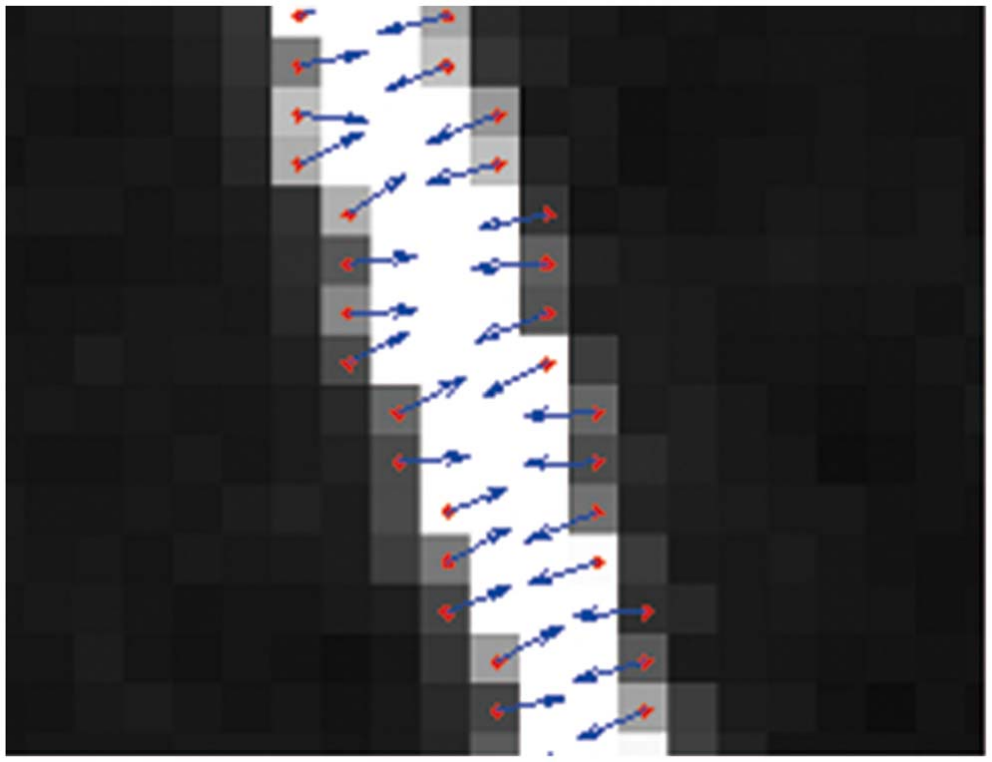
Determination the normal direction.

**Figure 9:**
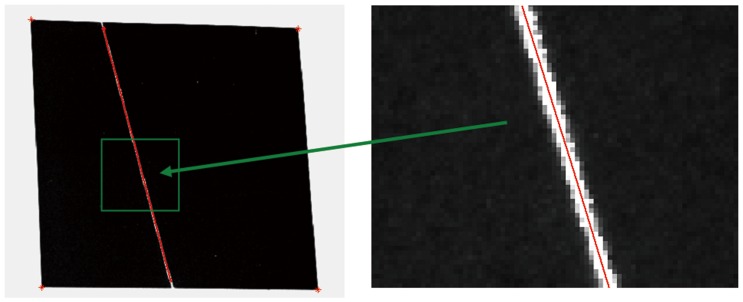
Detection of light stripe center.

1). The edge points of light stripe are detected in pixel-level by Sobel operator [23], as shown in Fig. 7;

2). The gray gradient direction of the edge points are calculated as the normal direction of light stripe curve, and the cross section of light stripe can be obtained along the normal direction, as shown in Fig. 8;

3). A closed solution for extracting light stripe center is derived based on spatial moment theory, and the sub-pixel coordinates of light stripe center can be obtained fast in all cross sections, as shown in Fig. 9.

Using the intrinsic parameters and the plane equation of the target, all sub-pixel coordinates of the light stripe centre can be projected into the camera coordinate frame. In this way, the sufficient control points can be got for projector calibration.

#### 2.3 Fitting the structured light plane

**Figure 10:**
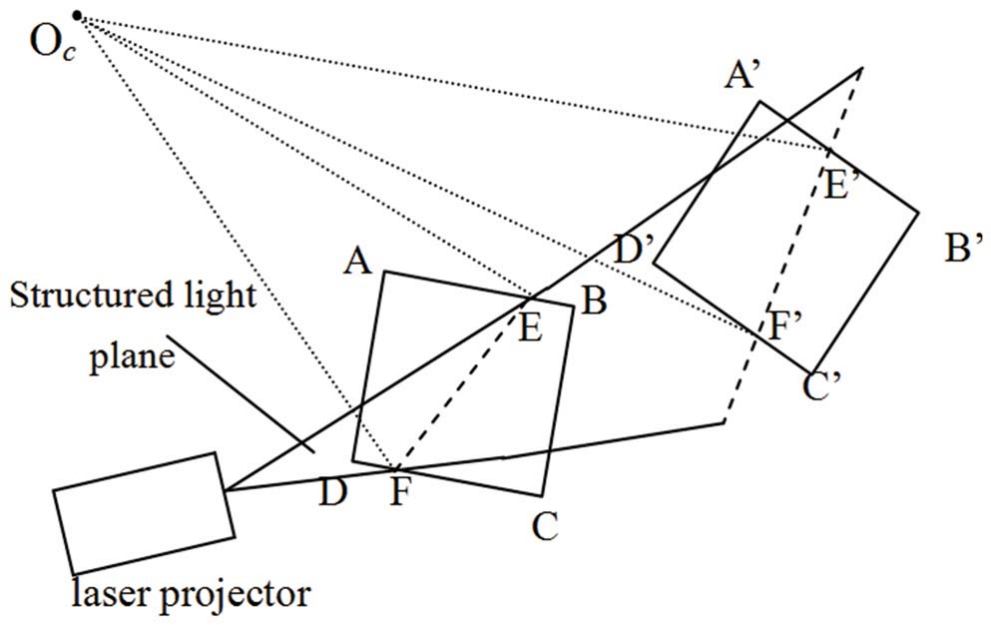
Fitting the structured light plane.



Given that the equation of the structured light plane is described by.
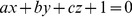
(7)where *a*, *b* and *c* are the unknown coefficients. To obtain the accurate equation of the plane, the following work is carried out: move the target in the space repeatedly, and then the multi-group non-collinear projection points of the light strip centers in the camera coordinate frame can be obtained as control points. As shown in Fig. 10, all the control points should locate in the structured light plane theoretically. Thus, the structured light plane can be determined by fitting the control points 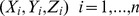, and the computation formula for solving equations is as follows:

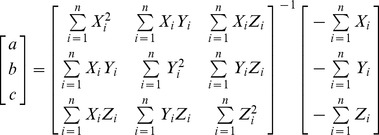
(8)Based on the intrinsic parameters and the light plane coefficients, the world coordinates of intersection points of the light plane with the world object surface can be solved.

## Experimental Studies

### 1. Calibration experiment

**Figure 11:**
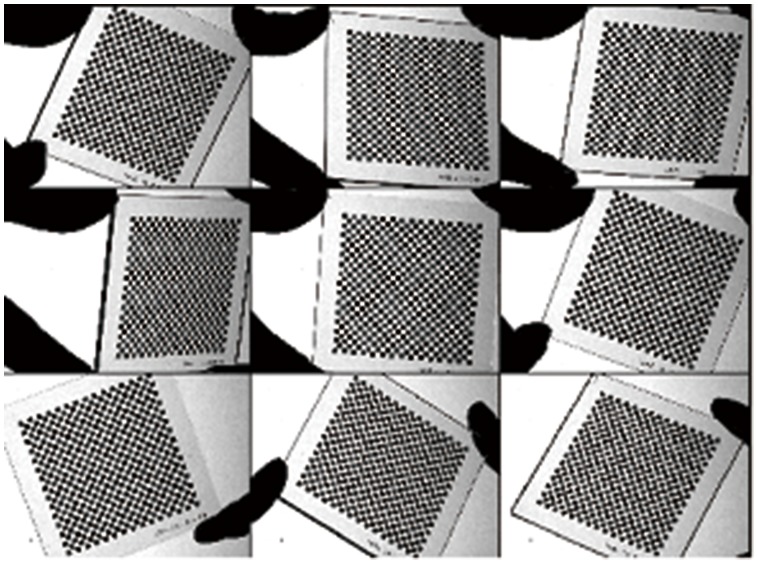
Calibration patterns for camera.

The structured-light sensor designed in this work consists of a CCD camera with a 25 mm lens and a laser plane projector (wave length 650 nm, line width <0.8 mm). In the experiments, the intrinsic parameters 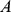 and 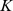
of the camera should be calibrated using the patterns from a checker board in advance. The precision of the grid on the checker board is 1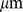. Nine patterns of the checker board are acquired by the camera at a spatial image resolution of 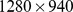 pixels, as shown in Fig. 11. Using these patterns, the camera model given in section 2 is calibrated, and the intrinsic parameters 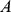 and 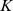 are obtained and listed in Table 3.

**Table 3:** Parameters of Zhang’s method.

Intrinsic parameters matrix	distortion coefficients	Average residuals
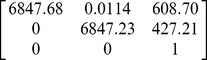	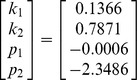	0.0033 (mm)

**Figure 12:**
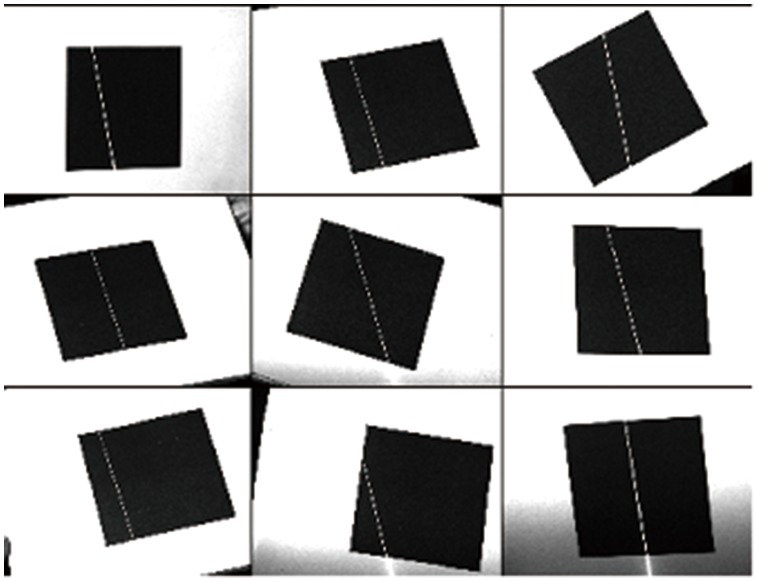
Calibration patterns for projector model.

When the camera calibration is completed, nine patterns of a target with a square pattern (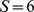
cm) are acquired by the camera, as shown in Fig. 12. Using the proposed method, the equations of the structured light plane can be solved:
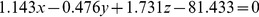
(9)


### 2. Accuracy tests

**Figure 13:**
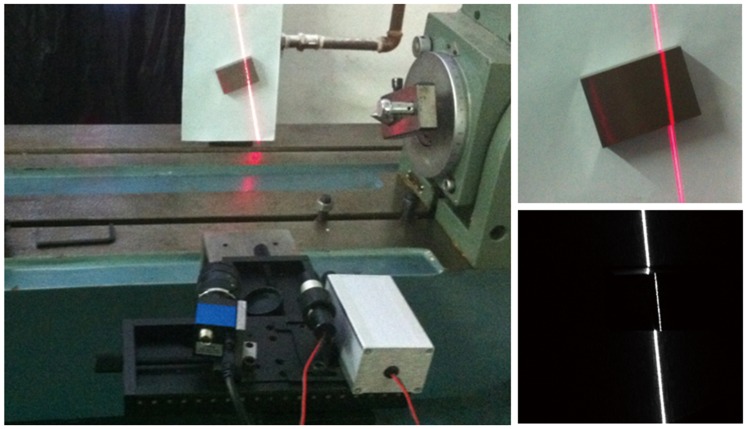
The imaging of gauge block.

**Figure 14:**
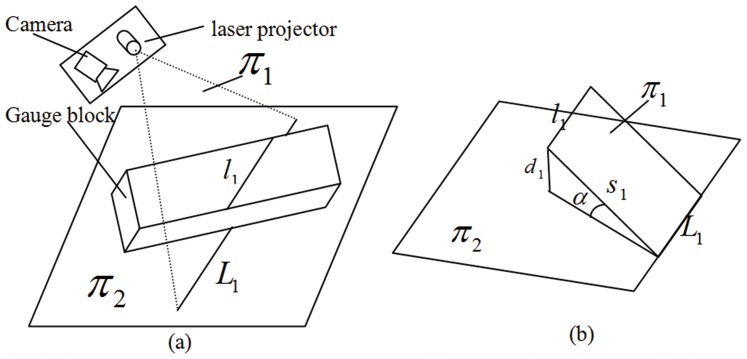
The schematic diagram of measurement.

To test the accuracy of line structured light vision system, the rectangular parallelopiped gauge blocks with known widths are measured using the system, and the measurement steps are as follows:

1). The light stripe is projected onto a gauge block, and the image of the light stripe is acquired by the camera, as shown in Fig. 13;

2). Two parallel lines 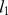 and 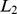 can be determined in the camera coordinate frame, as shown in Fig. 14(a);

3). Based on the spatial relationship in Fig. 14(b), the angle between the structured light plane 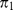 and the background plane 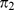 can be solved as
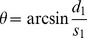
(10)where 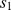 is the distance between the lines 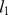 and 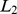, and 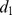 (the distance between the line 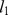 and the plane 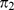) is the real width of gauge block.

Usually, to improve the accuracy of angle 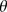, more than one gauge blocks are used to get the mean value in the test. With the help of the angle 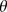, the system can be used to measure other gauge block’s width based on Eq. (10). Because every block’s width is known, the average absolute errors of measurement can be obtained by comparing with the real width, and they can show the error of the system.

**Figure 15:**
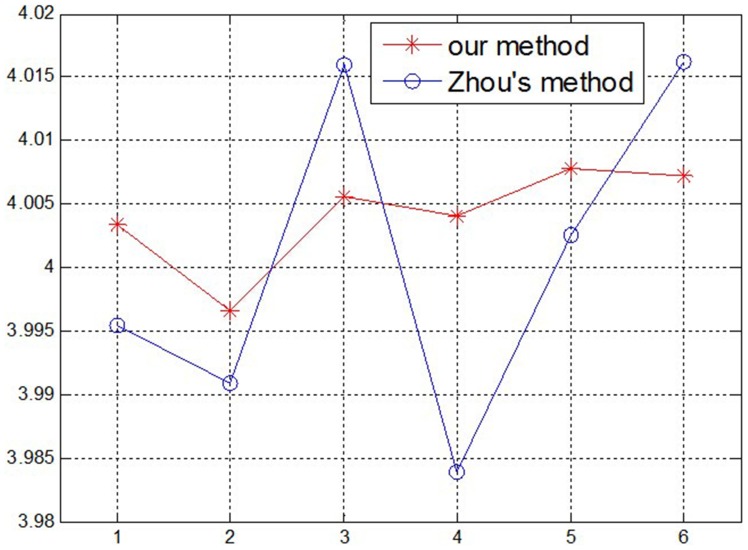
The measurement results of 4 mm Gauge block.

**Figure 16:**
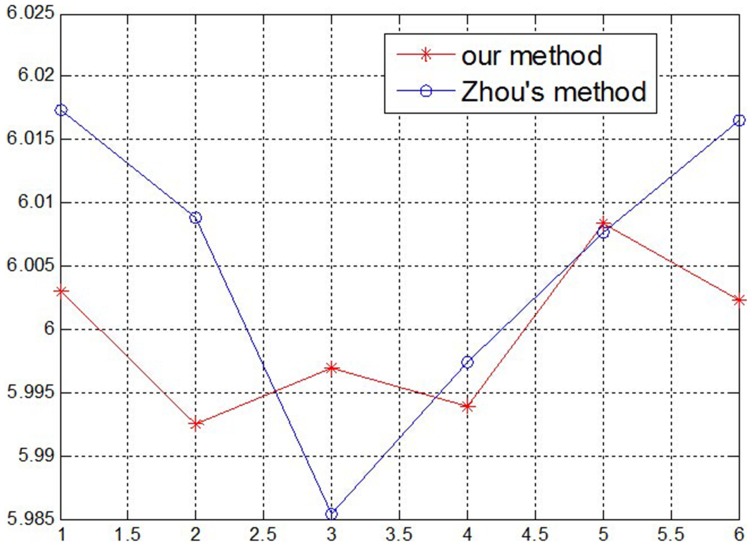
The measurement results of 6 mm Gauge block.

**Figure 17:**
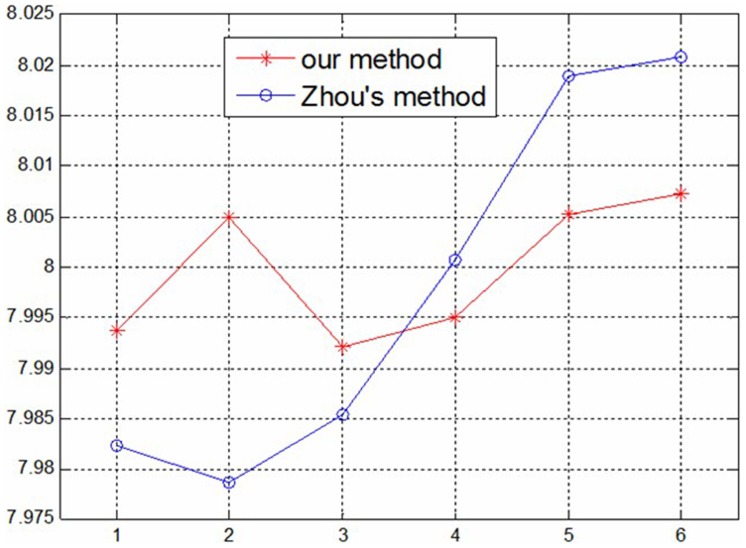
The measurement results of 8 mm Gauge block.

**Figure 18:**
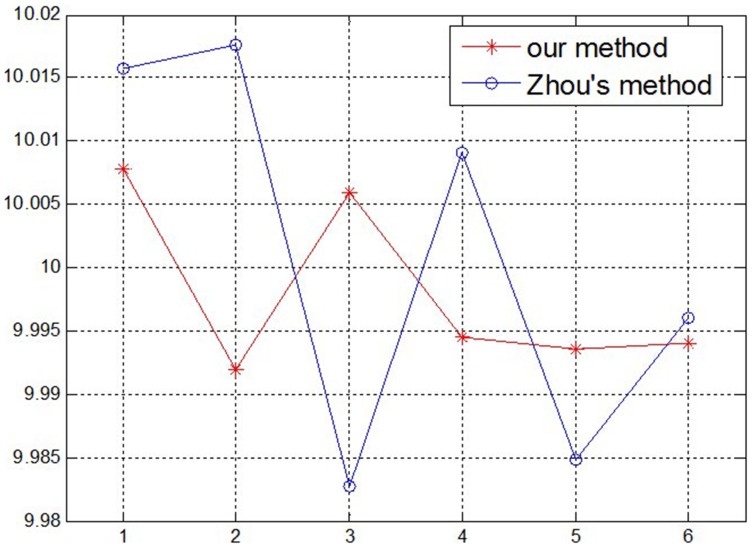
The measurement results of 10 mm Gauge block.

Using the above measurement method, the methods in this paper and Zhou’s [14] are compared: first, two group parameters of the structured light plane are obtained by using the two methods; then the same gauge block is measured respectively using the two group parameters with the help of the structured light system. In this test, four gauge blocks are measured using the system, and every block is measured six times, as shown in Fig. 15–18. According to the measurement results in Table 4 and 5, the measurement accuracy of the proposed method is higher than Zhou’s, which has proved that the parameters of structured light plane obtained by the proposed method is more accurate. In the Zhou’s method, since the control points are generated by intersecting of light stripe and limited grids in the target, a few control points can be obtained, and it is difficult to constrain the structured light plane better. In the proposed method, the planar target can be randomly moved, and all light stripe centers on the target image are extracted as the control points. More control points can achieve over-constraint of the structured light plane, which ensures the accuracy of the light plane equation.

**Table 4:** The measurement results of the proposed method (mm).

Gauge block	number of measurement times						Known width	Measurement errors
	1	2	3	4	5	6		
1	4.0034	3.9966	4.0056	4.0041	4.0078	4.0072	4	0.0053
2	6.0030	5.9925	5.9969	5.9939	6.0084	6.0023	6	0.0051
3	7.9937	8.0049	7.9921	7.9950	8.0052	8.0073	8	0.0061
4	10.0078	9.9919	10.0059	9.9945	9.9936	9.9941	10	0.0066

**Table 5:** The measurement results of Zhou’s method (mm).

Gauge block	number of measurement times						Known width	Measurement errors
	1	2	3	4	5	6		
1	3.9954	3.9909	4.0160	3.9839	4.0026	4.0162	4	0.0108
2	6.0174	6.0088	5.9854	5.9974	6.0077	6.0166	6	0.0113
3	7.9824	7.9787	7.9854	8.0008	8.0189	8.0208	8	0.0157
4	10.0158	10.0176	9.9827	10.0091	9.9848	9.9960	10	0.0132

## Discussion and Conclusion

A novel calibration method for a structured light vision system by viewing a planar target from unknown orientations is proposed in this paper. In the method, the planar target with a square pattern is used to generate sufficient non-collinear control points for structured light stripe vision. This method provides a high accuracy, and is low-cost and easy to use on-site. The experiments conducted on a real structured light vision system that consists of one camera and one single light stripe plane laser projector reveal that the proposed approach is of high accuracy and is practical in the vision measurement applications. The proposed approach greatly reduces the cost of the calibration equipment and simplifies the calibrating procedure.

## References

[1] Gao Y, Wang M, Zha ZJ, Tian Q, Dai Q (2011) Less is more: efficient 3-D object retrieval with query view selection. IEEE Transactions on Multimedia 13: 1007–1018.

[2] Gao Y, Wang M, Tao D, Ji R, Dai Q (2012) 3-d object retrieval and recognition with hypergraph analysis. IEEE Transactions on Image Processing 21: 4290–4303. 22614650 10.1109/TIP.2012.2199502

[3] Gao Y, Wang M, Ji R, Wu X, Dai Q (2014) 3D object retrieval with hausdorff distance learning. IEEE Transactions on industrial electronics 61: 2088–2098.

[4] Leandry I, Breque C, Valle V (2012) Calibration of a structured-light projection system: Development to large dimension objects. Optics and Lasers in Engineering 50: 373–379.

[5] Xu J, Xi N, Zhang C, Shi Q, Gregory J (2011) Real time 3D shape inspection system of automotive part based on structured light pattern. Optics and Laser Technology 43: 1–8.

[6] Kim MY, Ayaz SM, Park J, Roh YJ (2014) Adaptive 3D sensing system based on variable magnification using stereo vision and structured light. Optics and Lasers in Engineering 55: 113–127.

[7] Chen F, Brown GM, Song M (2000) Overview of the three-dimensional shape measurement using optical methods. J. Opt. Eng 39: 10–22.

[8] Guan T, Duan L Y, Yu J Q (2011) Real-time camera pose estimation for wide-area augmented reality applications. IEEE computer graphics and applications 31: 56–68. 10.1109/MCG.2010.23 24808092

[9] Guan T, Wang C (2009) Registration based on scene recognition and natural features tracking techniques for wide-area augmented reality systems. IEEE Transactions on Multimedia 11: 1393–1406.

[10] Tsai R Y (1987) A versatile camera calibration technique for high-accuracy 3D machine vision metrology using off-the-shelf TV cameras and lenses. IEEE Journal of Robotics and Automation 3: 323–344.

[11] Zhang Z (2000) A flexible new technique for camera calibration. IEEE Trans. on Pattern Analysis and Machine Intelligence 22: 1330–1334.

[12] Dewar R (1988) Self-generated targets for spatial calibration of structured light optical sectioning sensors with respect to an external coordinate system. Proc. Robots and Vision’88 Conf., 5.

[13] Huynh DQ (1999) Calibration a structured light stripe system: a novel approach. Int. J. Comput. Vis. 33: 73–86.

[14] Zhou F, Zhang G (2005) Complete calibration of a structured light stripe vision sensor through planar target of unknown orientations. Image and Vision Computing 23: 59–67.

[15] Wei Z, Cao L, Zhang G (2010) A novel 1D target-based calibration method with unknown orientation for structured light vision sensor. Optics & Laser Technology 42: 570–574.

[16] Duan F, Liu F, Ye S (2000) A new accurate method for the calibration of line structured light sensor. Chinese J. Sci. Instrum. 21: 108–110.

[17] Xu J, Douet J, Zhao JG, Chen K (2013) A simple calibration method for structured light-based 3D profile measurement. Optics & Laser Technology 48: 187–193.

[18] Xie ZX, Zhu WT, Zhang ZW (2010) A novel approach for the field calibration of line structured-light sensors. Measurement 43: 190–196.

[19] Zhou FQ, Cui Y, Peng B (2012) A novel optimization method of camera parameters used for vision measurement. Optics & Laser Technology 44: 1840–1849.

[20] JeanYB. Pyramidal implementation of the Lucas Kanade feature tracker description of the algorithm. Available from: http://www.vision.caltech.edu/bouguetj/index.html.

[21] Steger C (1998) An unbiased detector of curvilinear structures. IEEE Trans. Pattern Analysis and Machine Intelligence 20: 113–125.

[22] Sun QC, Hou YQ, Tan QC (2014) A Fast and Robust Detection Algorithm for Light Stripe Center. Submitted to Lasers in Engineering. (accepted/in press)

[23] Sobel I (1978) Neighbourhood coding of binary images fast contour following and general array binary processing. Computer Graphics and Image Processing 8: 127–135.

